# Acute laparoscopic and open sigmoidectomy for perforated diverticulitis: a propensity score-matched cohort

**DOI:** 10.1007/s00464-015-4694-8

**Published:** 2015-12-17

**Authors:** Sandra Vennix, Daniel J. Lips, Salomone Di Saverio, Bart A. van Wagensveld, Walter J. Brokelman, Michael F. Gerhards, Anna A. van Geloven, Susan van Dieren, Johan F. Lange, Willem A. Bemelman

**Affiliations:** 1 Department of Surgery, Academic Medical Center, Postbox 22660, 1100 DD Amsterdam, The Netherlands; 2 Department of Surgery, Erasmus MC University Medical Center, Rotterdam, The Netherlands; 3 Department of Surgery, Jeroen Bosch Hospital, ’s-Hertogenbosch, The Netherlands; 4 Department of Surgery, Hospital Maggiore, Bologna, Italy; 5 Department of Surgery, Sint Lucas Andreas Hospital, Amsterdam, The Netherlands; 6 Department of Surgery, OLVG Hospital, Amsterdam, The Netherlands; 7 Department of Surgery, Tergooi Hospital, Hilversum, The Netherlands; 8 Clinical Research Unit, Academic Medical Center, Amsterdam, The Netherlands

**Keywords:** Diverticulitis, Laparoscopy, Sigmoid resection, Perforated diverticulitis, Propensity score

## Abstract

**Background:**

Hartmann’s procedure for perforated diverticulitis can be characterised by high morbidity and mortality rates. While the scientific community focuses on laparoscopic lavage as an alternative for laparotomy, the option of laparoscopic sigmoidectomy seems overlooked. We compared morbidity and hospital stay following acute laparoscopic sigmoidectomy (LS) and open sigmoidectomy (OS) for perforated diverticulitis.

**Methods:**

This retrospective cohort parallel to the Ladies trial included patients from 28 Dutch academic or teaching hospitals between July 2010 and July 2014. Patients with LS were matched 1:2 to OS using the propensity score for age, gender, previous laparotomy, CRP level, gastrointestinal surgeon, and Hinchey classification.

**Results:**

The propensity-matched cohort consisted of 39 patients with LS and 78 patients with OS, selected from a sample of 307 consecutive patients with purulent or faecal perforated diverticulitis. In both groups, 66 % of the patients had Hartmann’s procedure and 34 % had primary anastomosis. The hospital stay was shorter following LS (LS 7 vs OS 9 days; *P* = 0.016), and the postoperative morbidity rate was lower following LS (LS 44 % vs OS 66 %; *P* = 0.016). Mortality was low in both groups (LS 3 % vs OS 4 %; *P* = 0.685). The stoma reversal rate after Hartmann’s procedure was higher following laparoscopy, with a probability of being stoma-free at 12 months of 88 and 62 % in the laparoscopic and open groups, respectively (*P* = 0.019). After primary anastomosis, the probability of reversal was 100 % in both groups.

**Conclusions:**

In this propensity score-matched cohort, laparoscopic sigmoidectomy is superior to open sigmoidectomy for perforated diverticulitis with regard to postoperative morbidity and hospital stay.

**Electronic supplementary material:**

The online version of this article (doi:10.1007/s00464-015-4694-8) contains supplementary material, which is available to authorized users.

The classic Hartmann’s procedure for perforated diverticulitis can be characterised by high morbidity and mortality rates [1, 2]. Nowadays, the treatment of has shifted towards less invasive procedures such as laparoscopic lavage, percutaneous drainage, or even conservative management for the milder cases with perforated diverticulitis [3–5]. As laparoscopic lavage has shown to be effective in 75 % of the patients with purulent perforated diverticulitis, superior to sigmoidectomy (e.g. Hartmann’s procedure or with primary anastomosis) with regard to morbidity and mortality in a randomised controlled trial, it is important to explore other less invasive treatment options besides the classic open Hartmann’s procedure [6].

The proven benefit of the laparoscopic approach in the elective setting might even be more pronounced in emergency sigmoidectomy than in the elective setting avoiding in particular abdominal wall complications, e.g. abdominal wound dehiscence, incisional hernia, and wound infection [7–9]. In a systematic review of 5 studies including 104 patients, acute laparoscopic sigmoidectomy for perforated diverticulitis has been shown to be feasible, but comparative studies are lacking [10]. Previously, laparoscopic surgery for acute peritonitis has been under debate due to theoretical concerns of increased bacteraemia and hypercapnia due to the pneumoperitoneum [11, 12]. However, more recent studies suggest even a protective role of the CO_2_ pneumoperitoneum with a reduced systemic inflammatory response, but similar bacterial translocation [13, 14].

In this propensity-matched cohort, we aim to show a reduction in morbidity and hospital stay following acute laparoscopic sigmoidectomy (LS) compared to open sigmoidectomy (OS) for perforated diverticulitis with generalised peritonitis.

## Methods

### Patients

This retrospective observational cohort consists of consecutive patients with perforated diverticulitis that were not included in the randomised Ladies trial in 28 Dutch teaching hospitals during a 3-year period [6]. Despite the low accrual rate, these patients did not differ in baseline from those randomised within the Ladies trial [6]. The patients were retrospectively identified using the hospital administration code for diverticulitis and acute abdomen, combined with a surgical intervention code to determine the inclusion rate of the Ladies trial. Only the patients requiring acute sigmoidectomy for perforated diverticulitis with purulent or faecal peritonitis have been included. Those with peritoneal lavage or enterostomy without resection have been excluded from analysis, as were those with disease located in other sections than the left colon or sigmoid and those with Hinchey I–II disease or coincidence of fistula. Within the cohort, patients with laparoscopic sigmoidectomy have been identified and were matched 1:2 with patients with open sigmoidectomy based on propensity scores. The term sigmoidectomy is used for both Hartmann’s procedure and resection with primary anastomosis; if only one of these two is addressed, the terms Hartmann and primary anastomosis are used. As only anonymous patient data were collected, no ethical approval was required under Dutch law.

### Outcomes

Data have been collected regarding age, gender, BMI (body mass index), American Society of Anesthesiologists (ASA) classification, prescription medication, history of diverticulitis, previous laparotomy, CT diagnosis, preoperative C-reactive protein (CRP) and white blood cell (WBC) count, acute physiology and chronic health evaluation-II (APACHE-II [15]) score, P-POSSUM (Portsmouth Physiology and Operative Severity Score for the enumeration of Mortality and Morbidity [16]) score, and interval from presentation at the emergency department to surgery. Perioperative data have been collected on the Mannheim peritonitis index (MPI) [17], Hinchey classification [18], diagnostic laparoscopy, conversion, intraoperative complications, duration of surgery, and the presence of a gastrointestinal surgeon (defined as a consultant-level surgeon specialised in colorectal or gastrointestinal surgery). Postoperative outcomes assessed were morbidity, scored as Clavien–Dindo ≥I or ≥IIIB [19], mortality, length of hospital stay, ICU admission, and surgical or percutaneous reinterventions. Long-term data were collected on last follow-up, mortality, stoma reversal, and incisional hernia.

### Cost analysis

An economical evaluation was performed to evaluate the costs of laparoscopic versus open sigmoidectomy up to 30 days postoperative or until discharge in the matched cohort. Direct medical costs were estimated using primary data on resource utilisation and included all surgical procedures, including reinterventions and radiological reinterventions, hospital ward stay, and ICU stay. Costs per patient were calculated by multiplying volumes of resources with unit costs. These costs were determined according to the Dutch guidelines of pharmacoeconomic research or based on the tariff of the Academic Medical Center, Amsterdam. Costs were expressed in Euros and inflated when necessary to 2012.

### Statistical analysis

Before matching, continuous variables were presented as mean with standard deviation (SD) or median with interquartile range (IQR) when appropriate. Discrete variables were presented as numbers of events with percentages. Univariate testing was performed using a *t* test or Mann–Whitney *U* test when the data were not normally distributed. Pearson’s Chi-squared or Fisher’s exact test was used for categorical and dichotomous data.

To control for potential confounders, a propensity score was generated for each patient from a multivariable logistic regression model based on baseline variables as independent variables with OS and LS treatment as a binary dependent variable. Those baseline variables in the univariate analysis with a *P* value <0.1 or otherwise of specific clinical interest were added to the model. Sufficient predictive value of the propensity score was defined as an area under the curve (AUC) of ≥0.7 and a Hosmer and Lemeshow (HL) test *P* ≥ 0.05.

Each laparoscopic case was matched with replacement to two open cases in a 2:1 ratio, with a caliper width of 0.20 standard deviation of the logit of the propensity score using R statistical software (version 2.13.1). The quality of the match was assessed by comparing the patient characteristics before and after matching as shown in eTable 1 and Table 1.

**Table 1:** Patient demographics in the propensity-matched cohort

	Laparoscopic sigmoidectomy *N* = 39	Open sigmoidectomy *N* = 78	*P* value
Age, years	56.2 (14.2)	56.4 (13.3)	0.930
Gender, male	14 (35.9)	24 (30.8)	0.593
BMI, kg/m^2^	25.3 (3.5)	27.1 (5.8)	0.883
ASA I	7 (22.6)	13 (23.6)	0.441
ASA II	12 (38.7)	29 (52.7)	
ASA III	11 (35.5)	11 (20.0)	
ASA IV	1 (3.2)	2 (3.6)	
Prescription medication	19 (48.7)	32 (43.2)	0.578
History of diverticulitis	7 (17.9)	14 (17.9)	1.000
Previous laparotomy	1 (2.6)	1 (1.3)	1.000
CT diagnosis	35 (89.7)	65 (85.5)	1.000
CRP level	158 (118)	166 (119)	0.450
WBC count	15.4 (8.9)	13.7 (6.2)	0.232
APACHE-II score	7.4 (5.0)	6.6 (4.2)	0.163
P-POSSUM predicted mortality (%)	9.3 (11.7)	10.5 (13.4)	0.935
POSSUM predicted morbidity (%)	67.7 (17.1)	67.5 (17.5)	0.575
Interval to surgery, hours	11 (6–48)	11 (6–25)	0.095
Gastrointestinal surgeon present	38 (97.4)	76 (97.4)	1.000
MPI score	19.2 (5.3)	18.3 (4.6)	0.236
Hinchey IV	8 (20.5)	13 (16.7)	0.608

Data are mean (SD), number (%), or median (interquartile range)

*BMI* body mass index, *ASA* American Society of Anesthesiologists, *CT* computed tomography, *CRP* C-reactive protein, *WBC* white blood cell, *APACHE*-*II* acute physiology and chronic health evaluation-II, *POSSUM PS* POSSUM—physiology score, *POSSUM OS* POSSUM—operative score, *MPI* Mannheim peritonitis index

Data in the matched data set were analysed using a generalised random block design for continuous variables and conditional logistic regression for categorical variables. A two-sided *P* value of <0.05 was considered statistically significant for all tests. Analyses were performed using IBM SPSS version 20.0 software and R version 2.13.1.

### Statistical power

According to the published literature on elective sigmoidectomy, a 15 % reduction in postoperative major morbidity (Clavien–Dindo grade IIIB or higher) is expected for laparoscopic sigmoidectomy (10 %) compared to open sigmoidectomy (25 %). A power of 0.478 is to be expected, using *α* = 0.05 and groups of 39 and 78 patients. Double the sample size would be required to gain a power (1-β) of 0.8 (78 and 156 patients) with a 15 % difference.

## Results

### Patient characteristics

**Fig. 1:**
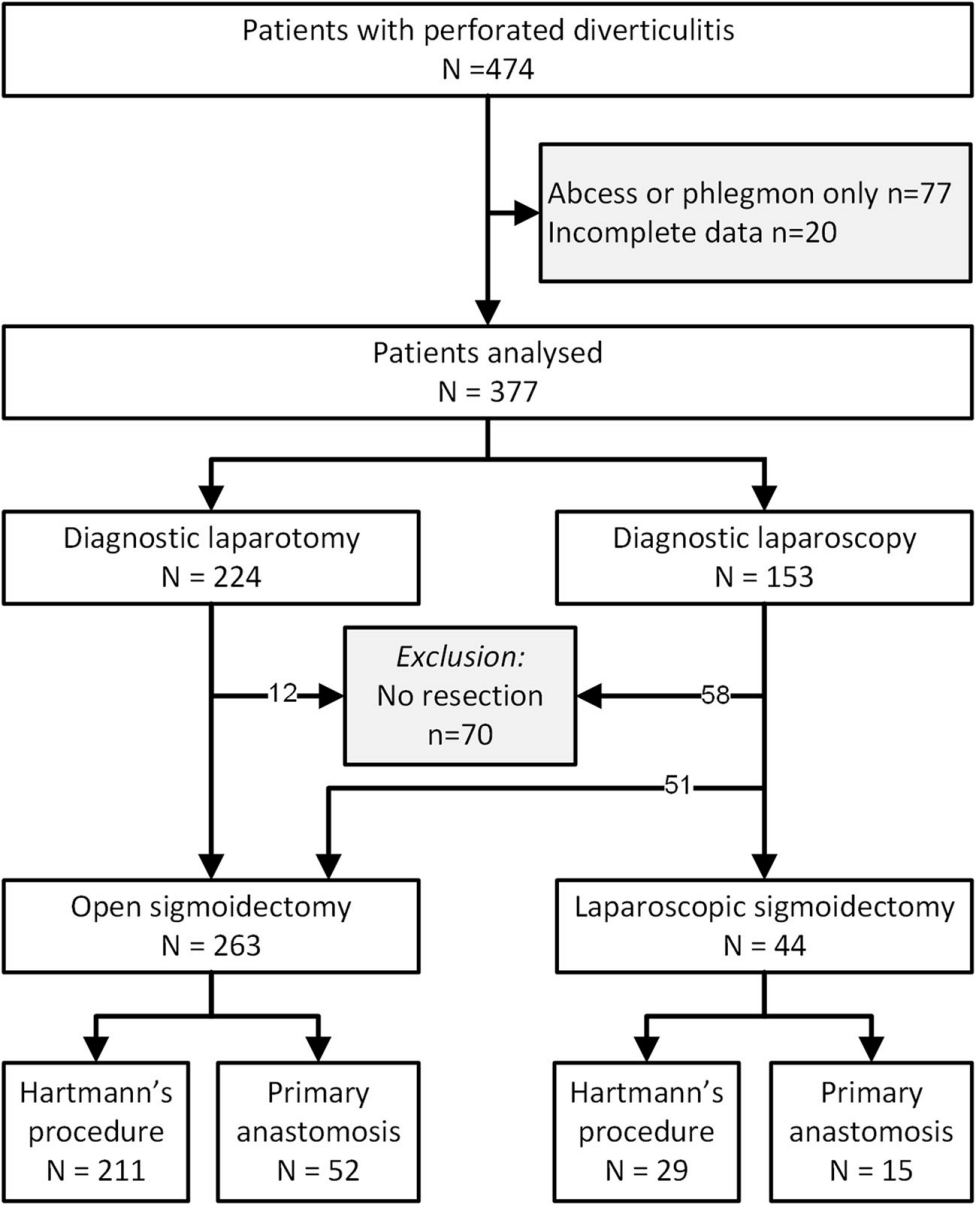
Patient flow chart

A total of 474 patients treated for perforated diverticulitis could be identified between July 2010 and July 2014 (Fig. 1). Of these, data were available for 377 patients with purulent or faecal peritonitis. A diagnostic laparoscopy (DLS) was performed in 153 (41 %) patients, of whom 58 did not undergo resection, 51 were converted to laparotomy, and 44 had laparoscopic sigmoidectomy. From the 224 patients with DL, another 12 were excluded because no resection was performed. In two of the 44 (4.5 %) patients in the LS group, laparoscopic resection was attempted, but converted due to adhesions and distended small bowel, and therefore insufficient exposure of the sigmoid perforation.

Patients with LS were identified in 19 out of the 28 hospitals, ranging between 1 and 9 patients each.

In univariate analysis (eTable 1), we found both patients’ age and CRP level to be predictive for the choice between LS and OS. Patients with faecal peritonitis and with a higher MPI score were more likely to undergo OS. Specialised gastrointestinal surgeons were more likely to perform LS.

In multivariate analysis with age, gender, previous laparotomy, preoperative CRP level, gastrointestinal surgeon, MPI, and Hinchey classification, only CRP, GI surgeon, and Hinchey classification were found to be predictors for LS (eTable 2). Using all variables from the multivariate analysis, a propensity score for LS or OS was calculated. Only MPI was not included in the propensity score as MPI calculation includes both age and Hinchey classification. The calculated propensity score had a sufficient predictive value with an area under the curve (AUC) of 0.772 (95 % CI 0.704–0.841; HL test, *P* = 0.309).

### Propensity-matched cohort

The baseline characteristics of the propensity-matched cohort are shown in Table 1. In a 1:2 matching strategy, 39 patients with laparoscopic sigmoidectomy were matched to 78 patients with open sigmoidectomy. Due to the replacements during the automatic matching process, the control group consisted of 59 unique patients with open sigmoidectomy and 19 duplicates. Following matching, no differences persisted in any of the matched variables as expected. In addition, no differences were found between the groups with regard to all other variables such as the ASA classification and preoperative POSSUM and APACHE scores. Sigmoidectomy was performed as Hartmann’s procedure in 66 % of patients and primary anastomosis in 34 %. Of these patients with primary anastomosis, 8/13 had a deviating ileostomy following LS compared to 12/27 in the OS group. The surgical duration for LS was longer with 127 min compared to 97 min for OS (*P* = 0.003) (Table 2).

**Table 2:** Surgical and short-term postoperative outcomes in the propensity-matched cohort

	Laparoscopic sigmoidectomy *N* = 39	Open sigmoidectomy *N* = 78	*P* value
Duration of surgery, minutes	127 (105–159)	96.5 (87–120)	0.003
Hartmann’s procedure	26 (66.7)	51 (65.4)	0.890
Primary anastomosis	13 (33.3)	27 (34.6)	
Ileostomy rate	8/13 (61.5)	12/27 (44.4)	0.597
Postoperative ICU admission	11 (36.7)	28 (50.0)	0.305
In-hospital mortality	1 (2.6)	3 (3.9)	0.685
In-hospital overall morbidity	17 (43.6)	51 (66.2)	0.016
In-hospital severe morbidity (>IIIB)	5 (12.8)	15 (19.5)	0.253
Reinterventions	5 (12.8)	15 (19.5)	0.739
Surgical reinterventions	2 (5.1)	7 (9.1)	0.485
Percutaneous reinterventions	3 (7.7)	10 (13.0)	0.419
Postoperative hospital stay, days	7 (5–13)	9 (7–14)	0.016

Data are mean (SD), number (%), or median (interquartile range). *ICU* intensive care unit. Severe morbidity defined as Clavien–Dindo ≥IIIB

Intraoperative complications occurred in 1 patient during LS and two patients during OS. Two patients had an intraoperative bleeding, and in one patient, the stapler donuts of the attempted primary anastomosis were incomplete and an end colostomy was created instead. Intraoperative blood loss was reported as <100 ml in 14 (74 %) patients in LS and 15 (42 %) in the OS (*P* = 0.117). Following acute surgery, 11 (37 %) LS patients and 28 (50 %) OS patients were admitted to the ICU.

### Postoperative outcomes

Laparoscopic sigmoidectomy resulted in a shorter hospital stay compared to OS (7 versus 9 days, *P* = 0.016). The in-hospital overall morbidity rate was lower following LS (*P* = 0.016). No significant difference was found with regard to in-hospital mortality and reinterventions as shown in Table 2. In-hospital mortality was reported in 1 (3 %) patient following LS and in 3 following OS (4 %). Overall morbidity was lower in the laparoscopic group. No difference was seen for surgical reinterventions (LS 5 % vs OS 9 %; *P* = 0.485) or severe morbidity (Clavien–Dindo ≥IIIB, LS 13 % vs OS 20 %; *P* = 0.253). A significant reduction in wound infections was found (LS 3 % vs OS 29 %; *P* = 0.009, eTable 3). Anastomotic leakage occurred in one patient following LS and was treated by relaparotomy and loop ileostomy.

In a subgroup analysis, laparoscopic and open Hartmann’s procedure were compared, showing 8 % (OS) versus 4 % (LS) mortality (*P* = 0.476), and severe morbidity occurred in 39 % (OS) versus 15 % (LS) (*P* = 0.037). Postoperative hospital stay was 12 (8–21) days versus 8 (5–15) days for open and laparoscopic Hartmann’s, respectively (*P* = 0.006). In the primary anastomosis group, the mortality and severe morbidity rate were 0 % in both the laparoscopic and open group. Postoperative hospital stay was 8 (7–9) days versus 7 (6–10) days for open and laparoscopic primary anastomosis, respectively (*P* = 0.391).

The calculated costs were lower for laparoscopic sigmoidectomy (mean difference € −8 336, 95 %CI € −16 113 to € 588; *P* = 0.031); these costs included only the direct costs for the primary hospital admission. For subgroups of costs, particularly the costs for ICU stay were five times higher for open sigmoidectomy (*P* = 0.022) (Table 3).

**Table 3:** Costs calculation based on short-term data only (Euro)

	Lap sigmoidectomy		Open sigmoidectomy		*P* value
	Units	Total costs	Units	Total costs	
Primary surgery	39	153,426	78	241,878	–
Days at hospital ward	355	172,328	846	410,674	0.108
Days at ICU	38	88,114	350	806,942	0.022
Percutaneous drainage	3	486	10	1619	0.418
Surgical reinterventions	3	9589	8	25,571	0.698
Total		423,943		1,486,684	
Total per patient, Euro	10,870 (4710–17,031)		19,209 (14,850–23,563)		0.031

Total per patient as mean (95 % confidence interval). Three surgical reinterventions in two patients, and eight reinterventions in seven patients

*ICU* intensive care unit

### Long-term outcomes

**Fig. 2:**
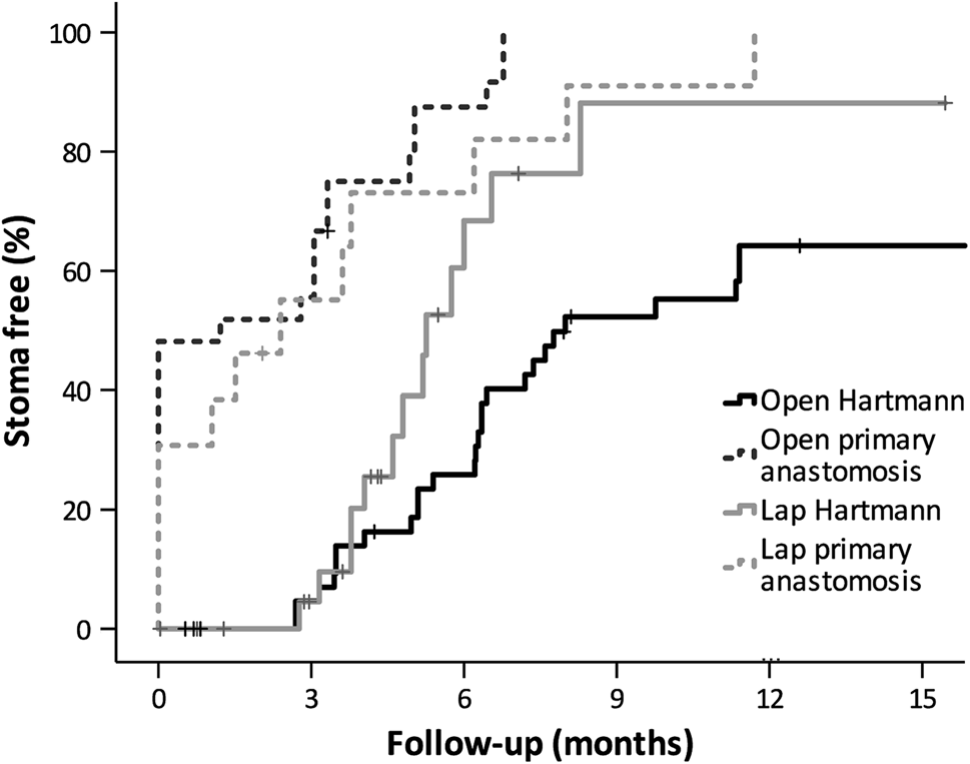
Probability of being stoma-free. Generated by Kaplan–Meier method. Log rank for all groups *P* < 0.001. Log rank for Hartmann’s lap versus open *P* = 0.019. Log rank for primary anastomosis lap versus open *P* = 0.272

The median length of follow-up was shorter in the LS group with 8 (IQR 5–12) months compared to 16 (IQR 7–28) months in the OS group (*P* < 0.001) as more patients had LS later in the study period (eTable 4). Stoma reversal was associated with Hartmann’s procedure or primary anastomosis and not with laparoscopic or open surgery in multivariable regression analysis (data not shown). Therefore, Kaplan–Meier analysis was performed for four groups: open Hartmann’s, open primary anastomosis, laparoscopic Hartmann’s, and laparoscopic primary anastomosis with probabilities of being stoma-free at 12 months of 0.64, 1.00, 0.88, and 1.00, respectively (*P* < 0.001, log rank test; Fig. 2 and eFigure 1). Colostomies in the laparoscopic group were reversed more often using the laparoscopic technique (12/13 LS, 4/28 OS; *P* < 0.001).

## Discussion

This first comparative study between open and laparoscopic sigmoidectomy shows laparoscopic sigmoidectomy for perforated diverticulitis to be superior to open sigmoidectomy with regard to morbidity and hospital stay. Laparoscopic sigmoidectomy is safe and feasible as shown by the low conversion rate and postoperative mortality that did not differ significantly compared to OS. The lower morbidity and hospital stay resulted in reduced costs per patient in the laparoscopic group. Stoma closure after Hartmann’s procedure occurred more often after a laparoscopic approach.

Up to now, only a few small non-comparative series have been described regarding laparoscopic sigmoidectomy for perforated diverticulitis [10]. Favourable results were described in these selected patients, especially in comparison with the older open sigmoidectomy series. Another series by Turley et al. [20] compared two groups of 67 patients in a propensity-matched cohort out of 1186 patients with emergency Hartmann’s procedure for diverticulitis. No statistically significant differences in postoperative morbidity (30 vs 25 %), mortality (4.5 and 3.0 %), and hospital stay (8 vs 6 days) were shown between open and laparoscopic surgery. A limitation of that study is the unclear indication for surgery in the included patients, which was not limited to Hinchey III and IV perforated diverticulitis.

In the EAES guidelines for emergency abdominal surgery, laparoscopic sigmoidectomy is described as a feasible option in experienced hands [21]. However, the cited paper by Zdichavsky et al. [22] describes a semi-acute series after failed medical management of low grades of perforated diverticulitis. Although a slightly different population, these series do support the feasibility of laparoscopic sigmoidectomy in a contaminated abdomen.

Although overall postoperative morbidity was higher following open sigmoidectomy, only the difference in wound infection rate was statistically significant and made up for the complete 20 % difference. However, even without the wound infections, the total number of complications was higher following open sigmoidectomy (16 vs 73 surgical events and 23 vs 96 total events in 39 and 78 patients).

Stoma reversal did not differ between LS and OS after primary anastomosis; the probability of reversal was 100 % in both groups, while 38 % of the patients after LS and 56 % after OS never had an ileostomy. This finding is not surprising, because closure of the defunctioning ileostomy can be done without laparotomy. The initial approach, either laparoscopic or open, does not affect the reversal rate for this reason [23, 24]. Following Hartmann’s procedure, the probability of reversal for LS was 87 % compared to 63 % for OS. These data are reflected in previous study on acute Hartmann’s procedure, with a reversal rate of 72 % after LS and 57 % after OS in less selected patient populations [23, 25].

The main concern regarding laparoscopic surgery in general peritonitis is the risk of damage to the distended and vulnerable small bowel. A recent systematic review reported 64 % success of laparoscopic treatment in 2005 patients with small bowel obstruction. About 10 % of the conversions were due to iatrogenic injury and 7.6 % due to inadequate exposure [26]. Even a small bowel diameter >4 cm was not considered to be a contraindication for laparoscopy [27].

This study used a propensity-matched design to evaluate differences between both groups. This design allows for a correction for bias introduced by selection of patients, improving the reliability of the presented outcomes. This design has been shown to provide similar treatment effects compared to randomised studies and therefore can be relied upon when randomised trials are not feasible [28].

The present study has some limitations. First, its retrospective and non-randomised design might have introduced selection and reporting bias. Although all patient records were fully searched for outcomes, the registration might be incomplete compared to proper prospective registration. Second, the small proportion of patients with laparoscopic sigmoidectomy is likely to be selected based on favourable patient or disease characteristics, in combination with surgeon’s preferences. The patients selected for laparoscopy had a lower preoperative CRP level and a non-significant difference in age and ASA classification compared to open sigmoidectomy. Although only patients with Hinchey III and IV disease and no Hinchey II disease have been included, these results apply especially to patients with similar characteristics to those in this matched cohort.

After diagnostic laparoscopy, in 44 patients the procedure was continued by laparoscopy, while 51 patients were converted to laparotomy. Due to the retrospective nature of this study, the reason and moment of conversion remain unclear due to lack of standardised reporting. Some were converted before the diagnosis was clear and others upon diagnosing perforated diverticulitis. In two patients, conversion was described after attempting laparoscopic resection, and therefore recorded as conversion within the laparoscopic group, and analysed according to intention to treat.

## Conclusions

In this propensity score-matched cohort, laparoscopic sigmoidectomy is superior to open sigmoidectomy for perforated diverticulitis with regard to postoperative morbidity and hospital stay. Although the groups are matched, the results should be interpreted with caution as the cohort consists of selected patients with more favourable baseline characteristics compared to the complete group and surgery was performed by experienced gastrointestinal surgeons.

## Electronic supplementary material

Below is the link to the electronic supplementary material.

Supplementary material 1 (DOCX 34 kb)
