# Supplementary material for: Acute laparoscopic and open sigmoidectomy for perforated diverticulitis: a propensity score-matched cohort
Source: Surg Endosc. 2015 Dec 17;30:3889–96. doi: 10.1007/s00464-015-4694-8 (PMC4992031; doi:10.1007/s00464-015-4694-8)
Supplement: Supplementary file 1 — Supplementary material 1 (DOCX 34 kb) [file 464_2015_4694_MOESM1_ESM.docx]

**Online only supplements**

**eTable 1.** Patient demographics in the cohort of 307 patients

**eTable 2.** Multivariate analysis for predicting laparoscopic or open surgery in the unmatched cohort

**eTable 3.** Short term detailed morbidity in the propensity matched cohort

**eTable 4.** Long term outcomes in the propensity matched cohort

**eFigure 1.** Probability of being stoma free for the patients with stoma

**eTable 1. Patient demographics in the cohort of 307 patients**

|  | **Laparoscopic Sigmoidectomy**  **N=44** | **Open Sigmoidectomy**  **N= 263** | ***P-*value** |
| --- | --- | --- | --- |
| Age, years | 56.2 (13.5) | 62.6 (13.9) | 0.005 |
| Gender, male | 30 (68.2) | 138 (52.5) | 0.053 |
| BMI, kg/m^2^ | 25.6 (3.9) | 27.0 (5.8) | 0.107 |
| ASA I  ASA II ASA III ASA IV | 8 (24.2)  13 (39.4)  11 (33.3)  1 (3.0) | 26 (16.5)  72 (45.6)  51 (32.3)  9 (5.7) | 0.672 |
| Prescription medication | 20 (47.6) | 152 (58.7) | 0.179 |
|  |  |  |  |
| History of diverticulitis | 7 (16.7) | 50 (19.1) | 0.709 |
| Previous laparotomy | 1 (2.3) | 26 (9.9) | 0.146 |
| CT diagnosis | 39 (90.7) | 217 (83.8) | 0.466 |
|  |  |  |  |
| CRP level | 156 (114) | 221 (142) | 0.002 |
| WBC count | 15.3 (8.7) | 14.7 (11.4) | 0.699 |
| APACHE II score | 7.0 (4.9) | 8.0 (4.7) | 0.208 |
| POSSUM PS score | 19.0 (5.7) | 20.7 (6.4) | 0.077 |
| POSSUM OS score | 19.3 (1.9) | 19.3 (1.9) | 0.996 |
| P-POSSUM predicted mortality (%) | 8.5 (11.3) | 11.2 (14.4) | 0.154 |
| POSSUM predicted morbidity (%) | 65.1 (17.8) | 69.5 (17.3) | 0.133 |
|  |  |  |  |
| Interval to surgery, hours | 11 (6-38) | 12 (6-47) | 0.787 |
| Gastrointestinal surgeon present | 42 (97.7) | 181 (69.9) | <0.001 |
| MPI score | 19.1 (5.4) | 21.1 (5.6) | 0.027 |
| Hinchey, IV | 10 (23.3) | 90 (34.7) | 0.022 |

Data are mean (SD), number (%) or median (IQR). POSSUM PS= POSSUM – Physiology Score. POSSUM OS= POSSUM Operative Score.

**eTable 2. Multivariate analysis for predicting laparoscopic or open surgery in the unmatched cohort**

|  | **Odds ratio** | **95% CI** | ***P*-value** |
| --- | --- | --- | --- |
| Age, years | 0.97 | 0.94-1.00 | 0.064 |
| Gender, male | 2.47 | 0.90-6.78 | 0.079 |
| Previous laparotomy | 2.85 | 0.35-23.15 | 0.327 |
| CRP level | 0.996 | 0.993-0.999 | 0.007 |
| Gastrointestinal surgeon present | 0.05 | 0.01-0.41 | 0.005 |
| MPI score | 1.11 | 0.98-1.27 | 0.097 |
| Hinchey IV | 3.52 | 1.07-11.63 | 0.039 |

OR= odds ratio. CI= confidence interval

**eTable 3.** **Short term detailed morbidity in the propensity matched cohort**

|  | **Laparoscopic Sigmoidectomy**  **N=39** | **Open Sigmoidectomy**  **N=78** | ***P-*value** |
| --- | --- | --- | --- |
| **Surgical morbidity** |  |  |  |
| Surgical reintervention | 2 (5.1) | 7 (9.1) | 0.485 |
| Abdominal sepsis | 2 (5.1) | 11 (14.3) | 0.153 |
| Anastomotic leakage | 1 (7.7) | 0 (0.0) | 0.144 |
| Incisional/stoma site hernia | 0 (0.0) | 2 (2.6) | 0.999 |
| Abdominal abscess | 4 (10.3) | 12 (15.6) | 0.453 |
| Postoperative bleeding | 3 (7.7) | 4 (5.2) | 0.567 |
| Postoperative ileus | 3 (7.7) | 13 (16.9) | 0.227 |
| Wound infection | 1 (2.6) | 22 (28.6) | 0.009 |
| Fistula | 0 (0.0) | 2 (2.6) | 0.999 |
|  |  |  |  |
| **Medical morbidity** |  |  |  |
| Respiratory insufficiency | 1 (2.6) | 7 (9.1) | 0.241 |
| Urinary tract infection | 2 (5.1) | 2 (2.6) | 0.488 |
| Pneumonia | 1 (2.6) | 5 (6.5) | 0.360 |
| Delirium | 3 (7.7) | 9 (11.7) | 0.505 |

**eTable 4. Long term outcomes in the propensity matched cohort**

|  | **Laparoscopic Sigmoidectomy**  **N=39** | **Open Sigmoidectomy**  **N=78** | ***P-*value** |
| --- | --- | --- | --- |
| Length of follow-up, months | 8 (5-12) | 16 (7-28) | 0.001 |
| Had stoma | 35 (89.7) | 64 (82.1) | 0.366 |
| Stoma reversed | 21/35 (60.0) | 43/64 (66.2) | 0.541 |
| Stoma interval, days | 140 (110-181) | 189 (105-231) | 0.119 |
| Laparoscopic colostomy reversal | 12/13 (92.3) | 4/28 (14.3) | <0.001 |
| Overall mortality | 3 (8.1) | 5 (6.7) | 0.876 |
| Incisional hernia | 2 (5.1) | 13 (16.7) | 0.063 |

**eFigure 1. Probability of being stoma free for the patients with stoma**


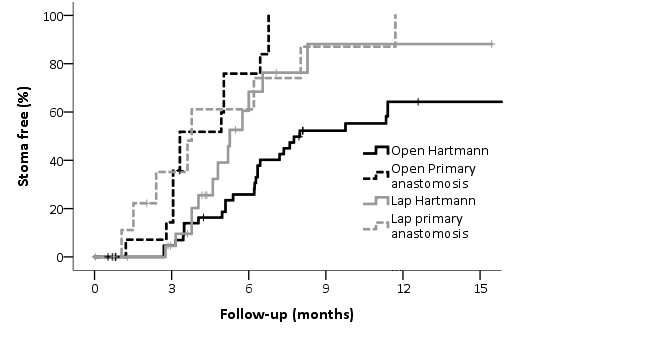


Generated by Kaplan-Meier method. Log rank for all groups P<0.001.

Log rank for Hartmann’s lap vs open P=0.019

Log rank for Primary anastomosis lap vs open P=0.544
